# An *in vitro* Assessment of the Haemodynamic Features Occurring Within the True and False Lumens Separated by a Dissection Flap for a Patient-Specific Type B Aortic Dissection

**DOI:** 10.3389/fcvm.2022.797829

**Published:** 2022-03-17

**Authors:** Liam Morris, Paul Tierney, Niamh Hynes, Sherif Sultan

**Affiliations:** ^1^Galway-Mayo Institute of Technology, Galway, Ireland; ^2^Galway Medical Technology Centre, Department of Mechanical and Industrial Engineering, Galway-Mayo Institute of Technology, Galway, Ireland; ^3^Medical and Engineering Technology Centre, Department of Mechanical and Industrial Engineering, Galway-Mayo Institute of Technology, Galway, Ireland; ^4^Lero – Science Foundation Ireland Research Centre for Software, Galway-Mayo Institute of Technology, Galway, Ireland; ^5^CÚRAM, National University of Ireland, Galway, Ireland; ^6^Western Vascular Institute, Department of Vascular and Endovascular Surgery, University College Hospital Galway, Galway, Ireland; ^7^Department of Vascular and Endovascular Surgery, Galway Clinic, Royal College of Surgeons in Ireland, Doughiska, Ireland

**Keywords:** aortic dissection, *in vitro* testing, phantom model, true and false lumens, blood pressure, flow patterns, dissection flap

## Abstract

One of the highest mortality rates of cardiovascular diseases is aortic dissections with challenging treatment options. Currently, less study has been conducted in developing *in vitro* patient-specific Type B aortic dissection models, which mimic physiological flow conditions along the true and false lumens separated by a dissection flap with multiple entry and exit tears. A patient-specific Stanford Type B aortic dissection scan was replicated by an in-house manufactured automatic injection moulding system and a novel modelling technique for creating the ascending aorta, aortic arch, and descending aorta incorporating arterial branching, the true/false lumens, and dissection flap with entry and exit intimal tears. The physiological flowrates and pressure values were monitored, which identified jet stream fluid flows entering and exiting the dissection tears. Pressure in the aorta’s true lumen region was controlled at 125/85 mmHg for systolic and diastolic values. Pressure values were obtained in eight sections along the false lumen using a pressure transducer. The true lumen systolic pressure varied from 122 to 128 mmHg along the length. Flow patterns were monitored by ultrasound along 12 sections. Detailed images obtained from the ultrasound transducer probe showed varied flow patterns with one or multiple jet steam vortices along the aorta model. The dissection flap movement was assessed at four sections of the patient-specific aorta model. The displacement values of the flap varied from 0.5 to 3 mm along the model. This model provides a unique insight into aortic dissection flow patterns and pressure distributions. This dissection phantom model can be used to assess various treatment options based on the surgical, endovascular, or hybrid techniques.

## Introduction

Aortic dissections have one of the highest mortality rates when compared to other cardiovascular diseases (1). Aortic dissections have challenging treatment options (1) and are the only cardiovascular disease that has no improvement in survival rates over the last four decades (2). The incidence of aortic dissection ranges from 5 to 30 cases per million people per year, depending on the prevalence of risk factors. Each year in Europe, an estimated 16,000 people are diagnosed with descending thoracic aortic pathology-complicated diseases. Although the disease is uncommon, its outcome is frequently fatal, and many patients with aortic dissections die before presentation to the hospital or prior to diagnosis (3, 4). Aortic dissections originate either at (1) the ascending aorta near the aortic valve or at (2) the descending aorta just distal to the origin of the left subclavian artery. The former is termed as Stanford Type A, and the latter, Stanford Type B (3), with approximately one-third of all aortic dissection cases attributed to acute Type B aortic dissections (5). Both the classified types of aortic dissections are caused by a tear between the intima and media layers creating true and false lumens, occurring anywhere along the human aorta which can affect some or the entire aorta from the aortic valve right down to the aortic bifurcation (6, 7). This tear creates a dissection flap comprised of intima, thin layer of media, and multiple intimal injury sites facilitating flow communication between the true and false lumens (8). During the past two decades, a variety of treatment options are available for aortic dissections. In general, Type A aortic dissections are treated surgically while medical management or endovascular stent grafts are used to treat Type B dissections (9). However, despite recent advances in medical, surgical, and endovascular treatments, this disease remains a formidable clinical challenge with high hospital mortality rates of 20% pre-admission mortality and 30% inhospital mortality, with many chronic Type B dissections developing complications (10). There are no reported differences in the long-term survival rates between Stanford Type A and Type B dissections (11).

Computational and experimental modelling can assist in understanding the haemodynamic phenomena that occur within aortic dissections. Both Schlicht (12) and Rudenick et al. (13) assessed pressure and velocity profiles within flexible straight dissection phantom models, under pulsatile flow conditions. A haemodynamic study was also conducted within an *in vitro* patient-specific Type B aortic dissection phantom model under steady (14) and pulsatile (15) flow conditions with undocumented stiffness or compliance values. Zadrazil et al. (16) performed steady flow computational and experimental simulations on an idealised Type B aortic dissection without branching vessels. Patient-specific computational studies incorporating branching vessels along a Type B dissection have previously been reported (17, 18). Pirola et al. (18) also compared their computational analysis with four-dimensional (4D) MRI. Bonfanti et al. (19) conducted pulsatile *in silico* and *in vitro* analysis on the patient-specific Type B aortic dissection models. At present, no fluid-structure interaction (FSI) studies have been published on aortic dissections (20). Few *in vitro* haemodynamic studies have been completed on aortic dissection models. There are currently no reported experiment models incorporating branches along the aortic dissection and patient-specific intimal tears. All documented aortic dissection models are limited to rigid walls. The addition of the aortic branches can promote further skewing of the flow with a spiral flow effect.

Aortic dissections are difficult to replicate due to their split lumen configuration and dissection tears. The majority of thin-walled flexible arterial phantom models, manufactured at present, are mostly comprised of single-lumen vessels replicating, for example, cerebral, coronaries, and aortic aneurysms with some models including layers for mimicking thrombus or lesions. However, very few experimental aortic dissection models exist which incorporate a true and false lumen separated by a dissection flap comprising of multiple intimal tears.

There is a requirement for aortic dissection simulator rigs to mimic physiological flow conditions through patient-specific thin-walled flexible aortic dissection models. Such test systems would allow for the haemodynamic analysis and the assessment of various treatment options based on the surgical, endovascular, or hybrid techniques. As far as the authors are aware, there is no patient-specific thin-walled aortic dissection model comprising of branching vessels and the true geometry of entry and exit tears, connected to a flow simulator capable of replicating the haemodynamic effects along a dissection model, assessing the flow communication through the dissection flap based on medical image datasets.

The main aim of this study was to (1) replicate a thin-walled flexible Type B aortic dissection model with branching vessels based on medical images and (2) assess the haemodynamic effects along this aortic dissection model with particular emphasis on the effects that the dissection flap has on flow patterns and pressure distributions within the true and false lumens.

## Materials and Methods

### Virtual Model

**FIGURE 1:**
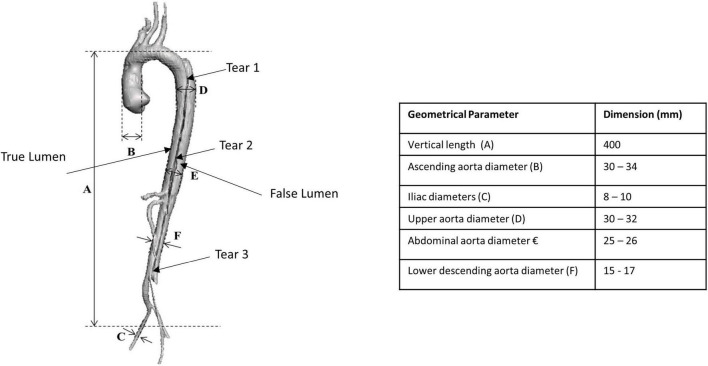
Three-dimensional generated patient-specific Type B aortic dissection model showing the dimensions of the various geometrical parameters.

A set of anonymised medical images in Digital Imaging and Communications in Medicine (DICOM) format were acquired from a CT scanner comprising of one patient-specific Stanford Type B aortic dissection case. This case was of a 30-year-old woman obtained from the Multi-Flow Modular (MFM) Global Registry hosted by the Western Vascular Institute. The open-source software, 3D Slicer, was used to generate a virtual three-dimensional (3D) model of the patient-specific aortic dissection comprising of the ascending aorta, aorta arch, descending aorta, iliac vessels with associated branching and dissection flap with three initial tears as shown in Figure 1.

### Phantom Model

**FIGURE 2:**
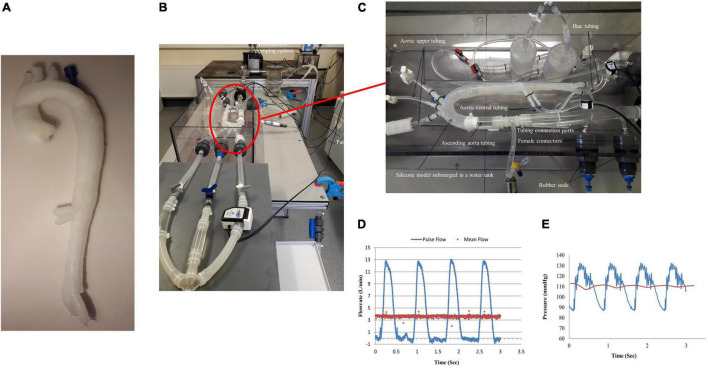
**(A)** Manufactured thin-walled flexible Type B aortic dissection model, **(B)** experimental flow rig, **(C)** zoomed in of attached aortic dissection model within the test rig, **(D)** measured inlet flowrate along the ascending aorta, and **(E)** measured inlet pressure within the ascending aorta.

A thin-walled flexible model was manufactured by the lost-wax process. Previous *in vitro* models for simulating blood flow through a single lumen within the coronaries (21, 22), cerebral (23–27), and aortic aneurysms (28–30) were previously replicated in our laboratory. An in-house automatic injection system was designed and manufactured in order to control the injection process. This aortic dissection model would be difficult to inject manually due to its split lumen configuration, thin walls, length (aortic valve to iliac vessels), and the entry/exit tears. The manual injection of a dissection model would lead to voids along the flap, which could not be easily filled in later. The automated injection moulding system controlled the feed rates and time delays for injecting silicone mixtures into different moulds and improved the quality of the models by eliminating the majority of bubbles (<5%) during the injection process. Previous manual injection methods had a reject/fallout rate of 55%, producing uneven wall thicknesses with air bubbles. A translucent two-part silicone elastomer Elastosil M4641 (Wacker Chemie AG, Germany) mixed in a ratio of 10:1 by weight, with 5% silicone fluid (Dow Corning, United Kingdom), was injected into a series of inner and outer mould cavities to create a thin-walled flexible Stanford Type B aortic dissection (Figure 2A). The Young’s modulus of this mixture was 1.2 MPa as determined by testing dog bone samples (BS ISO 37:2005, type 2) in a uniaxial tensile testing machine (Instron 5544, United Kingdom), equipped with 10N static load cell.

### Biosimulator

The pulsatile flow was replicated using a linear actuator (Aerotech, United Kingdom) that displaced a piston pump connected with two check valves. A steady flow circuit was set up in series with the pulsatile circuit using a direct drive pump (RD-05HV24, Iwaki Direct Drive Pump, Japan) capable of a maximum flowrate of 6 L/min and 9 m maximum head. The direct drive pump was controlled by a DC power supply. This in-line steady flow circuit allowed a non-positive flowrate waveform to be generated by superimposing the steady flow line on the pulsatile flow circuit. A commercially available Blood Mimicking Fluid (BMF, Model 046, CIRS, United States) was used in this system. It simulated the acoustic and physical characteristics of the blood, thus providing a stable and reliable fluid for Doppler studies. The temperature of the blood fluid replica was maintained constant at 37°C with the use of a heating unit (Julabo Ltd., United Kingdom). This heating unit provided continuous stirring and mixing of the fluid mixture. The inlet flowrates were measured by an ultrasonic flowmeter (TS410 plug-in module, Transonic, United States) with a flow sensor (25PXN Inline Flow Sensor, Transonic, United States) having an inner diameter of 25.4 mm. The flow sensor had a resolution of 1 ml/min for a scale of ±25 L/min for steady and pulsatile flows. The pressure transducer (Model FPG, R.D.P. Electronics Ltd., UK, with a range of 5 PSIG and output: 9.9556 mV/V) was used to measure pressures. A ∅1.2-mm (O.D.) angiographic access catheter (Terumo, Europe) was connected into the false lumen port comprising of the pressure transducer (R.D.P. Electronics Ltd., UK), to record pressures along the false lumen.

The outer wall displacements were monitored and recorded with a vision system connected to a CCD camera (Dalsa 4M30, Dalsa Corporation), with a frame rate of 30 frames per second, and a 4 MP resolution with attached Schneider Enlarger lens (aperture F 2.8). A circular fluoroscope light (CFVI Model 10, Coherent) positioned around the camera’s lens was applied to give the correct image brightness and contrast. The flow patterns, velocity profiles, and inner flap motion were visualised in real-time using the LOGIC-e Oxygen Care Ultrasound machine (GE Healthcare, IL, United States) and a VF13-5 128 Element Linear Array transducer probe, with a frequency range of 5–13 MHz. Figures 2B,C show the *in vitro* test rig. Figure 2D shows the generated inlet flowrate with a mean flowrate of 3.4 L/min, and Figure 2E shows the inlet pressure within the ascending aorta. Table 1 shows the % outflows for the various arterial branches.

**TABLE 1:** Flowrates through the various arterial branches.

Arterial branching	% outflow	Flowrate (L/min)
Innominate	10	0.333
Left common carotid	10	0.333
Left subclavian	10	0.333
Celiac	10	0.333
Superior mesenteric	10	0.333
Left and right renals	20	0.666
Left common iliac	15	0.500
Right common iliac	15	0.500

**FIGURE 3:**
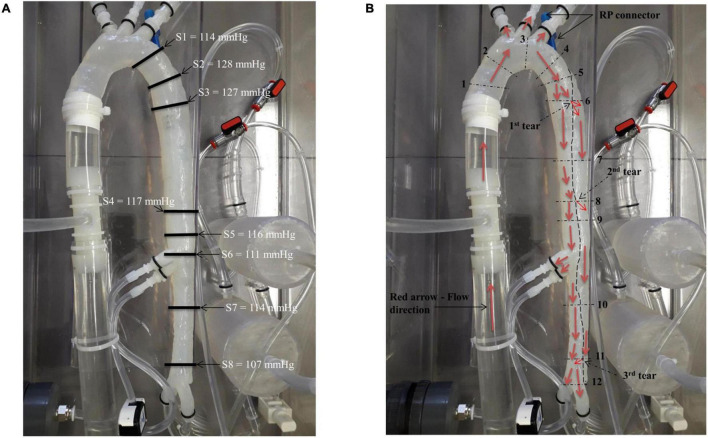
**(A)** Location of the pressure readings measured along the model and **(B)** locations of the flow patterns visualised along the model.

Pressure measurements and visualising various flow patterns were selected along the descending aorta as shown in Figure 3. These selected regions were in accordance with other clinical reporting standards (31).

## Results

### Geometrical Check

The aortic dissection model was scanned with an in-house Fluoroscopy X-ray machine [Ziehm Vision R Fluoroscope (Mobile C-arm), Germany]. The diameter sizes were analysed in Adobe Photoshop^®^ CS5 software package (Adobe, United States). There was a 1.3–2.4% difference in geometry between the 3D virtual model and the manufactured phantom model.

### Pressure Measurements

**FIGURE 4:**
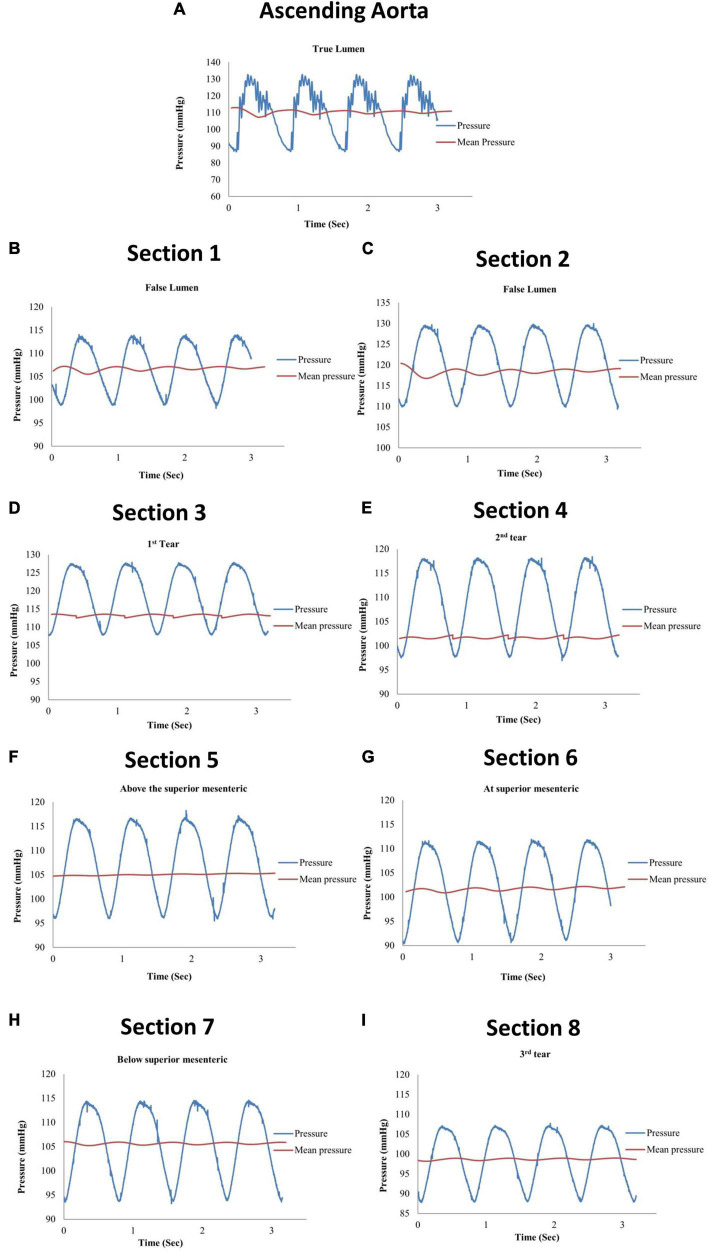
Pressure measurements for **(A)** the ascending aorta (true lumen) and **(B–I)** all eight sections along the false lumen.

Eight pressure readings were taken along the model including three dissection tear openings as shown in Figure 3A. Section 1 was measured directly by the pressure transducer, while Sections 2–8 were measured by the catheter attached to the pressure transducer within the false lumen. Figure 4 shows the pressure measurements for the ascending aorta (true lumen) and all eight sections along the false lumen. The pressure in the aorta’s true lumen region was controlled at 125/85 mmHg for systolic and diastolic values. The false lumen pressures differed in each region, ranging from a peak systolic value of 130 mmHg to a minimum diastolic value of 87 mmHg. It was found that the peak systolic pressure values (130 mmHg) at the top of the false lumen region had similar systolic pressure values in the true lumen. At the same location, the diastolic values increased from 87 to 110 mmHg in the false lumen. The false lumen pressures were lower than the true lumen pressures. At the first dissection tear, pressure values decreased slightly in the false lumen region by 2 mmHg in both systolic and diastolic values from 130 to 128 mmHg and 110 to 108 mmHg, respectively. This signifies that the flowrates were similar in both regions at this point of the aorta. At the second tear, pressure values dropped again in the false lumen to 118 mmHg systolic and 98 mmHg diastolic values. At the celiac trunk and superior mesenteric arteries, pressure values dropped by approximately 5 mmHg for systolic and diastolic values in the false lumen, while increasing again by 5 mmHg below both arteries. For the third and final tear, a noticeable drop in pressure of 8 mmHg was determined in the false lumen for both diastolic and systolic pressure readings.

### Flow Patterns Along the Patient-Specific Aorta

The patient-specific silicone model was monitored along 12 sections as shown in Figure 3B by the dashed lines. The direction of the flow is shown by arrows.

### Sections 1, 2, and 3

**FIGURE 5:**
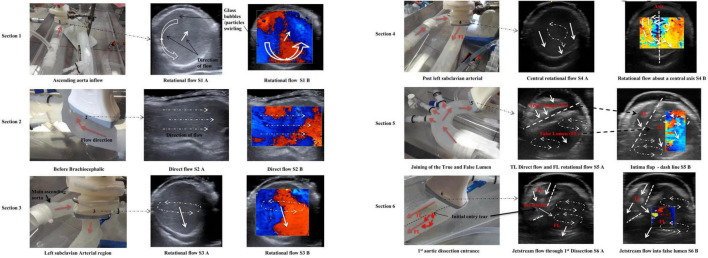
Monitored flow patterns by ultrasound for Sections 1–6.

Along the ascending aorta, there was a counterclockwise rotational flow pattern as viewed from the inlet (Figure 5 – Section 1 – S1 A) that travelled toward the upper branching vessels. The ultrasound colour Doppler (Figure 5 – S1 B) showed this rotational flow pattern with red and blue regions identifying flow travelling toward and away from the transducer.

The transducer probe was positioned along the centre line on the anterior side, showing a spiral flow as visualised by the colour Doppler ultrasound (Figure 5 – S2 B).

### Section 3

With the transducer probe positioned at the left subclavian artery “Section 3,” the fluid flow pattern rotated toward the centre of the vessel. The white solid arrow signifies the fluid direction. There was rotational flow from the posterior to the anterior side.

### Section 4

This section was 10 mm downstream from Section 3. Two (double) vortex flow patterns were observed as shown in Figure 5 (S4 A, S4 B).

### Section 5

This section was at the proximal end of the aortic dissection and proximal to the first tear. There were two vortexes in the false lumen region with recirculating regions. Both vortexes are located close to the internal adventitia wall (Figure 5 – Section 5).

### Section 6

Section 6 was along the first dissection tear measuring approximately 8 mm in length and located 30 mm from the top of the false lumen. There was a jet stream travelling across the tear, creating a large singular vortex motion within the false lumen. The dashed lines represent the intima flap with entry dissection (Figure 5 – Section 6).

### Section 7

**FIGURE 6:**
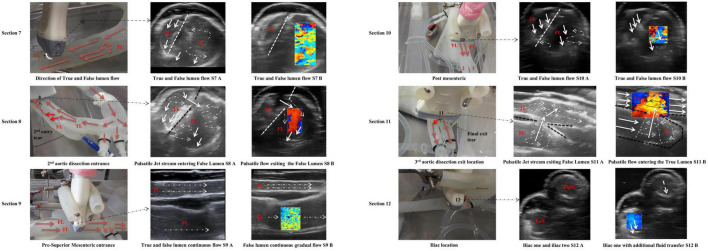
Monitored flow patterns by ultrasound for Sections 7–12.

Section 7 was between the first and second tears (Figure 6, Section 7). The flow within the true lumen is skewed toward the posterior wall. The false lumen had a singular vortex spinning toward the centre and parallel to the intima flap (Figure 6 – dashed line – S7 A). Figure 6 (Section 7 – S7 B) shows the mixing of the blood mimicking fluid as shown by the mosaic of colours.

### Section 8

Section 8 was across the second dissection tear located 110 mm from the top of the false lumen. The tear was approximately 5 mm in length and 3 mm in width. There were multiple vortices visualised within the false lumen (Figure 6 – Section 8). Possible reasons for these multiple vortices are due to a small split tear in the intima flap at the second tear. There was an increase in flowrates across the second tear, due to a combined flowrate between the first and second tears.

### Section 9

The velocity through the true lumen was much quicker than that of the false lumen region due to the narrowing of the true lumen (Figure 6 – S9 A, S9 B).

### Section 10

Section 10 was distal of the superior mesenteric artery; the true lumen had no recirculation regions while the false lumen had slower fluid flow with one vortex at the base of the vessel and a smaller vortex along the side of the intima flap (Figure 6 – S10 A, S10 B).

### Section 11

The ultrasound video imaging distinguished geometrical parameters for the third dissection hole in the vicinity of the region (Figure 6 – S11 A, S11 B). This third and final dissection tear were located 300 mm distally of the descending aorta’s false lumen. This exit dissection tear/hole measured 5 mm in diameter and was positioned at the very base of the false lumen region. For this particular tear, the fluid flow was exiting the false lumen and re-entering the true lumen. The flow through the third dissection hole was similar to the entry flow of the second dissection hole. One particular difference between the second entry dissection hole and the third was that the exit tear velocity shifted the true lumen flow against the artery wall (Figure 6 – S11 A, S11B).

### Section 12

This section defined the flowrate exiting both iliac arterial vessels. The flowrate on the iliac left side was exiting quicker than the iliac on the right side, as monitored by the ultrasound colour Doppler (Figure 6 – S12 B).

**FIGURE 7:**
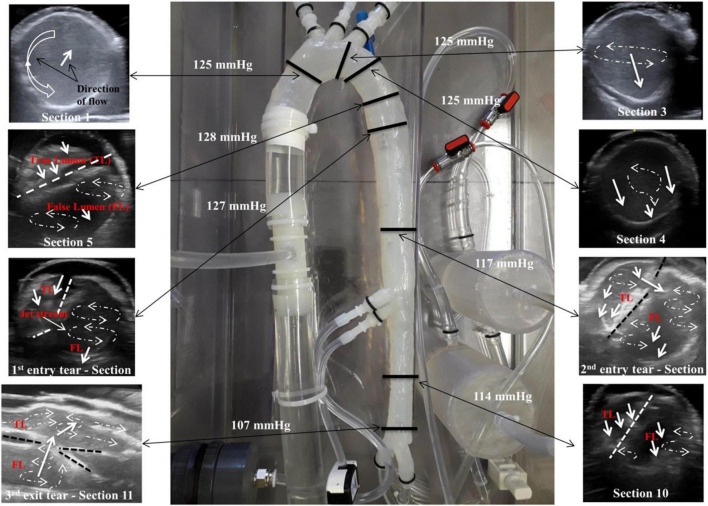
Flow patterns and peak pressure values in the false lumen.

Figure 7 shows the flow patterns and peak pressure values in the false lumen.

### Flap Movement

**FIGURE 8:**
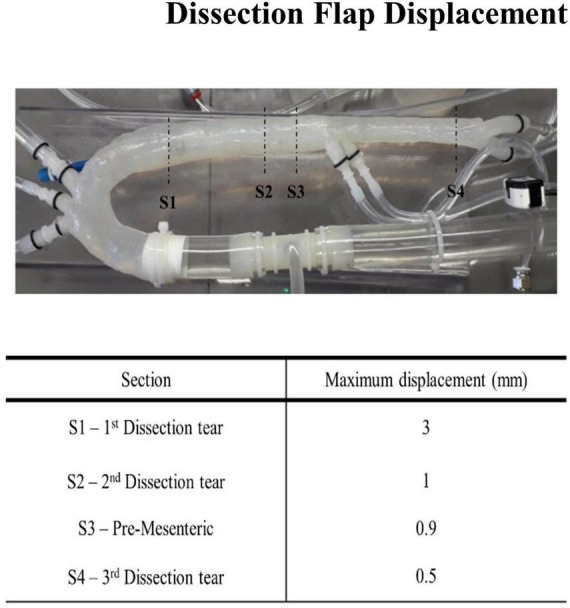
Dissection flap displacement.

The movement of the flap was assessed in four sections as shown in Figure 8. The displacement of the flap varied from 0.5 to 3 mm. The largest displacement occurred at the first aortic dissection tear. This was possibly due to the large dissection entry tear at the upper proximal section of the aorta. The final two dissections had displacement values ranging between 0.5 and 1 mm. This displacement value of 1 mm was consistent prior to the superior mesenteric region. The smallest displacement occurred after the third dissection. The intimal flap was slightly curved toward the false lumen for all stages of the cardiac cycle.

### Velocity and Flowrate Measurements Along the True/False Lumens and Across the Tears

**FIGURE 9:**
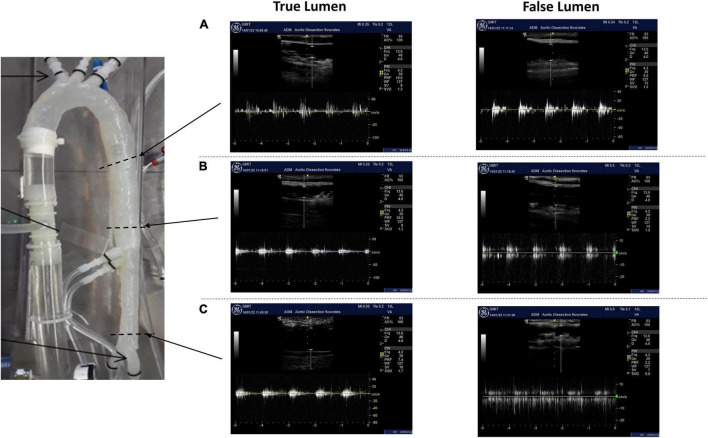
The average velocity profiles measured by Pulse Wave Doppler (PWD) along three sections of the aortic dissection model between **(A)** the first and second tears, **(B)** the second tear and superior mesenteric, and **(C)** the third tear and iliac vessels.

**FIGURE 10:**
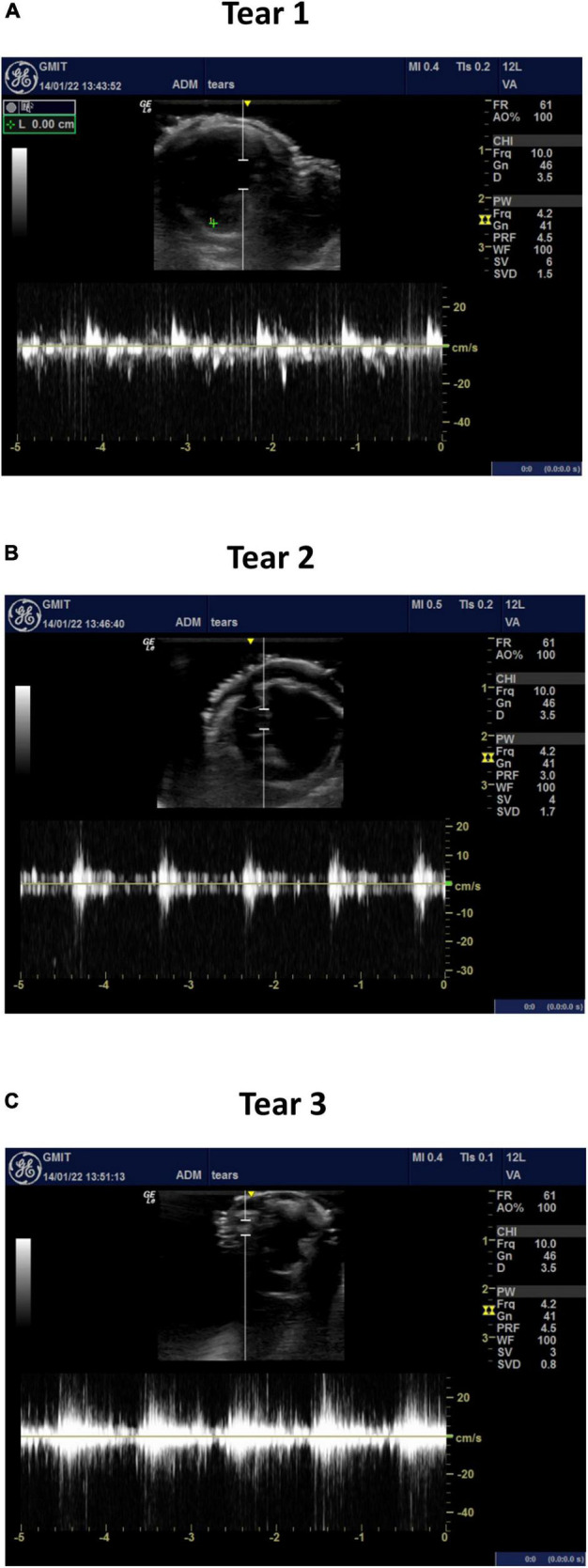
The average velocity profiles measured by PWD across **(A)** tear 1, **(B)** tear 2, and **(C)** tear 3.

The average velocity profiles measured by the ultrasound machine are shown along three sections of the aortic dissection model (Figure 9) and across the tears (Figure 10). The Pulse Wave Doppler (PWD) mode was applied. The Doppler sample volume (gate) was adjusted to completely encompass the vessel and tears. The Doppler sample volume was positioned parallel to the flow, and the transducer was positioned on the anterior side. The average velocity profiles across the vessels were measured between the first and second tears (Figure 9A), between the second and third tears (Figure 9B), and between the third tear and the iliac vessels (Figure 9C). There was a higher velocity magnitude along the true lumen when compared to the false lumen. The inlet velocities entering the false lumen peaked at ∼15 cm/s (Figure 10A) for the first tear and ∼11 cm/s (Figure 10B) for the second tear and re-entered the true lumen through the third tear with a maximum recorded velocity of ∼11 cm/s. The average estimated flowrates through tears 1 and 2 into the false lumen were 0.08 L/min (2.6% of total inlet flow over one cardiac cycle) and 0.05 L/min (1.6% of total inlet flow over one cardiac cycle), respectively. The peak systolic flowrates entering the false lumen through tears 1 and 2 were 0.25 and 0.1 L/min, respectively.

## Discussion

As far as the authors are aware, this is the first flexible thin-walled patient-specific aortic dissection model that included the full human aorta with branching vessels and intima tears, based on medical images. In particular, branching vessels were incorporated along the aortic dissection with patient-specific entry and exit tear geometries. No other reported experimental model has incorporated these patient-specific features. This *in vitro* model is a much-improved representation of the *in vivo* scenario that has not been experimentally replicated before. The geometrical accuracy of previously reported *in vitro* models is not documented. The phantom model presented in this study was scanned along different planes by a Fluoroscopy X-ray machine and compared against the original CT-scanned datasets. There was less than a 3% geometrical difference between the phantom model and CT-scanned datasets.

Physiological pressure values were determined for the true and false lumens. It is straightforward to take true and false lumen pressure measurements intraoperatively. However, this could only be carried out at the time of repair and could never be justified to be undertaken explicitly for the purposes of research. Therefore, getting patient-specific measurements in a patient who is under surveillance and not scheduled for repair would not be possible. To put this into context, intervention for patients with Type B aortic dissection is only indicated when they have complications. Such complications may include false lumen aneurysm formation, malperfusion (visceral, spinal, and lower limb), refractory pain, refractory hypertension, and rupture, and the majority of patients (57% based on the International Registry of Acute Aortic Dissection data) are treated medically (i.e., no intervention) (32).

*In vivo* studies have shown the existence of anticlockwise flow rotation in the descending aorta during systole (33, 34) similar to our observed flow patterns. Several different types of vortex patterns occurred within our phantom model commencing at the false lumen. A vortex can shield the surrounding blood flow causing platelet activation causing thrombus formation (35), which leads to arterial wall hypoxia, increased local inflammation resulting in localised wall weakening, and subsequent rupture (36). Karmonik et al. (37) observed significant aneurysmal dilation within a chronic dissection case and believed that this was due to thrombus formation. To the authors’ knowledge, these flow patterns and pressure values have not been determined before in a Type B aortic dissection model with arterial branching.

The intimal flap displacements measured within our model were in agreement with *in vivo* observed intimal flap motions of 0.5 ± 0.2 mm (38) and a range of 1.8–10.2 mm (39). Our findings were lower than that of Birjiniuk et al. (15), who experimentally obtained a maximum displacement of 14.3 ± 0.5 mm. A possible discrepancy in this displacement, with the displacement reported in our study, is that the model developed by Birjiniuk et al. (15) had an intimal flap thickness of 0.5 mm, which was thinner than our flap, and there could have been a greater pressure variation that was undocumented between the true and false lumen regions, in their study. The intima flap motion can be affected by the prognosis of late or chronic dissection tears, where the flap motion is more rigid or stiffer in nature, due to the progression of the disease (40). The wall thickness of the intimal flap may have an influence on the flap motion throughout the model.

The accurate calculation of the flowrates along the true and false lumens was not possible using the ultrasound machine. The PWD mode measures only the velocity, and the flowrate is found by multiplying the area by the velocity obtained across the vessel. The area is normally calculated by measuring the distance between the gates and assuming a circular cross-section. The flowrate is simply found by multiplying the area by the velocity obtained across the vessel. The cross-sections along both true and false lumens are of a half-moon shape and not circular in shape. For accurate flowrate measurements, PWD should be measured where the vessel is relatively circular (41). Hoyt et al. (42) evaluated five commercially available duplex ultrasound machines by three blinded experienced users within an *in vitro* test system comprising of a 6-mm inner diameter straight tube. They found an underestimation of the true flowrate for the flowrates exceeding 350 ml/min, except for one system that overestimated these values. They acknowledged the simplicity of their test setup, and further measurement imprecisions would occur within an animal model. Van Canneyt et al. (43) demonstrated, within an arteriovenous fistula model, that the PWD-based flowrate estimates are subject to a high degree of inaccuracy. This further demonstrates the difficulty in acquiring accurate flowrates from duplex ultrasound, especially for the patient-specific aortic dissection cases. The PWD mode measures in 1D and only captures the velocity component in the direction of the beam (43). The PWD captures the axial flow and not the resultant flow through the vessel caused by the spiral flow effects that were shown to occur within our model. Chen et al. (44) computationally simulated pulsatile flow within a patient-specific Type B dissection model and reported 25.4% of the inlet flow travelled through two entry tears into the false lumen over the cardiac cycle. This is a sixfold increase in flow travelling into the false lumen when compared to our findings. However, their study did not report the tear area, and this may explain this sixfold difference. Cheng et al. (45) simulated computationally eight patient-specific Type B aortic dissection cases. Their study found 6–82% of the systolic flow travelling through the tears into the false lumens for a wide variation of primary tear areas of 10–599 mm^2^. Over 50% of the systolic flow travelled into the false lumen for large enter primary tear areas of greater than 240 mm^2^. Much lower primary tear areas (∼10 mm^2^) have less than 10% of the systolic flow travelling entering the false lumens, similar to our study, which has a low primary tear area of 27 mm^2^. The maximum velocities along the main trunk before the first tear were 12 cm/s while along the true lumen the velocities varied from 40 cm/s (distal of the first tear) to 20 cm/s (distal of the third tear and proximal to the iliac vessels). This increase in velocity along the true lumen when compared to the main trunk proximal to the first tear was due to a reduction in the true lumen cross-sectional area. There was an increase of 25% in first tear velocity when compared to the main trunk velocity proximal to the first tear with a reduction of 8% in velocity for the second and third tears. The velocities along the true lumen exceeded the first tear velocity by 33–166%. This was due to the low tear area that reduced the systolic flow travelling into the false lumen. The exact phase difference between the aortic velocities and tear velocities could not be determined. Only one transducer could be positioned at any one time, and the ultrasound system could not be triggered. It was clear from the video evidence that the maximum tear velocities occurred during the systolic phase.

A patient-specific mould and an inner core model were created using a rapid prototyping machine. The automated injection moulding system assisted in injecting the patient-specific mould to produce the model. The patient-specific model was injected at a consistent feed rate with a silicone mixture using three injection ports on the mould, which is difficult to achieve manually. It was paramount to inject the patient-specific model on a first-time basis, due to the number of labour hours involved in printing and assembling the inner core and outer mould. The injection system provided complete control in injecting the silicone mixture into the patient-specific mould and minimised any unwanted variables (voids/bubbles) in the manufacturing process.

There remains much debate on the optimal management of aortic dissection. There has been increased use of endovascular techniques due to evidence suggesting that early endovascular management of Type B aortic dissection with thoracic endografting, although not enhancing survival, promotes aortic remodelling, which may, in turn, reduce the need for aortic reintervention (INSTEAD Trial and Adsorb Trial). However, the endovascular management of aortic dissection is not suitable in all patients due to anatomical constraints, in particular adequate proximal landing zones of the normal aorta to provide enough seal. Additionally, in the acute phase, endografting is associated with a risk of retrograde Type A dissection, whereas, in the chronic phase, endografting may be compromised by a small true lumen which becomes difficult to open due to the stiff uncompromising septum. Patient-specific phantom models may help to determine the feasibility of endovascular interventions in situations in which anatomical constraints are difficult to adequately quantify.

Other limitations to endovascular interventions in Type B dissections are the unknown long-term durability of stent grafts, which is of particular concern in younger patients. However, of greater concern is the adverse effects of aortic endografts on cardiac function. Current stent grafts have biomechanical properties that are several orders of magnitude stiffer than the native aorta. The materials used in TEVAR devices enhance the durability and reduce the risk of type IV endoleaks, but they stiffen the aorta. The aortic compliance serves a critical function to reduce the impedance and workload of cardiac ejection (46). Preclinical (30, 47–49) and preliminary clinical (50) studies have reported on acute stiffening of the aorta following TEVAR, resulting in acute elevated pulse pressure, hypertension, reduced coronary perfusion, and eventually heart failure (47, 48).

The lack of suitability of TEVAR devices for more proximal aortic applications has been demonstrated by the CORE group of investigators (51). They performed a computational study on eight patients and confirmed the deleterious late consequences of increased *in vivo* impedance and stiffness mismatch after TEVAR on left ventricular (LV) remodelling and coronary artery perfusion. They used a computational modelling workflow that enabled the quantification of LV Stroke Work from non-invasive imaging and pressure data. They demonstrated increases in LV mass and an increased requirement for intensive antihypertensive therapy to control blood pressure after TEVAR. Our pulsatile phantom model offers the potential to test aortic endograft stiffness relative to the specific aorta into which it is planned to be implanted and serves as an interesting test bed for the development of future devices with more favourable compliance, which can restore the functionality of the aorta to that of the non-diseased state and so preserve cardiac function (30). This model setup can alter physiological flow parameters from resting to exercise for pre- and post-operative testing. This would be an advantage that would not be easily performed in the clinical environment or simulated computationally, especially for turbulent flow and flexible wall conditions. Current computational models do not include flexible walls and tear flap motion. This setup is a pre-operative surgical planning tool, which would allow for various surgical scenarios and medical device types to be tested. Replicating the *in vivo* tear geometries would assess the performance of stent diverter systems such as the multilayer flow modulator stent for reducing or eliminating flow into the false lumen. It can also be used to validate computational models for various physiological flow scenarios. Further studies will be evaluated within our newly acquired Philips Azurion image-guided therapy platform capable of automated 3D motion capturing and flow evaluation. The novel manufacturing method outlined in this study can be applied to replicate Type A dissections.

There are a few limitations to this study. Only one subject case was tested, and the reported results may be difficult to generalise. There was no validation against *in vivo* measured data. The results reported in this study are unique to this patient-specific model geometry under the imposed physiological test parameters as would be the case for any patient-specific model. However, similarities in flow patterns and intimal flap motion were observed with other published *in vivo* studies providing confidence in the validity of our observed findings. Cheng et al. (45) further emphasised the requirement of incorporating septum motion within computational models. The septum motion will affect the pressures within the true/false lumens and flow distribution through the tears, which is currently absent from reported computational simulations. We have developed an established process that can be readily applied to other patient-specific models and clinical scenarios.

## Conclusion

An automatic injection system is required to manufacture thin-walled flexible dissection models. The pressures within the false lumen were lower than the true lumen along the descending aorta. Vortex flow patterns were found within the true and false lumen sections. The intimal flap showed movement from top to bottom of the false lumen, with a displacement ranging from 0.5 to 3 mm. Such a simulator would provide a surgical training platform and assessment of procedure/device performance. This would provide a valuable test facility that would assist clinicians on the optimum treatment option.

## Data Availability Statement

The raw data supporting the conclusions of this article will be made available by the authors, without undue reservation.

## Ethics Statement

The studies involving human participants were reviewed and approved by MFM Global Registry hosted by the Western Vascular Institute. The patients/participants provided their written informed consent to participate in this study.

## Author Contributions

All authors listed have made a substantial, direct, and intellectual contribution to the work, and approved it for publication.

## Conflict of Interest

The authors declare that the research was conducted in the absence of any commercial or financial relationships that could be construed as a potential conflict of interest.

## Publisher’s Note

All claims expressed in this article are solely those of the authors and do not necessarily represent those of their affiliated organizations, or those of the publisher, the editors and the reviewers. Any product that may be evaluated in this article, or claim that may be made by its manufacturer, is not guaranteed or endorsed by the publisher.
